# Integrative Analysis of Cellular Senescence-Related Genes Identifies FOLR1 as a Novel Tumor Suppressor and a Potential Therapeutic Target in Lung Adenocarcinoma

**DOI:** 10.3390/cancers18091330

**Published:** 2026-04-22

**Authors:** Fei Wang, Chang Xie, Min Zhang, Xiangyang Wu, Xinqi Sun, Yan Li, Zhibing Ming

**Affiliations:** 1 Department of Cardiovascular Surgery, Southeast University Affiliated Nantong First People’s Hospital, Nantong 226001, China; 5301333@ntu.edu.cn (F.W.); 13814657769@163.com (M.Z.); 2 Department of Vascular Surgery, Affiliated Nantong Clinical College of Nantong University, Nantong First People’s Hospital, Nantong 226001, China; xiechang2001@stmail.ntu.edu.cn (C.X.); 2531320276@stmail.ntu.edu.cn (X.W.); 3 Department of Vascular Surgery, Southeast University Affiliated Nantong First People’s Hospital, Nantong 226001, China; 213211160@seu.edu.cn

**Keywords:** lung adenocarcinoma, cellular senescence, FOLR1, prognostic model, tumor immune microenvironment

## Abstract

**Background:** Cellular senescence is a key regulatory mechanism in tumor initiation and progression, influencing cancer development through modulation of the cell cycle, the immune microenvironment, and inflammatory responses. However, the molecular characteristics and potential clinical value of senescence-related genes in lung adenocarcinoma (LUAD) have not been systematically elucidated. This study aimed to comprehensively characterize the expression patterns, molecular subtypes, and prognostic significance of cellular senescence-related genes in LUAD, and to identify key regulatory determinants. **Methods:** Transcriptomic data of cellular senescence-related genes were obtained from The Cancer Genome Atlas (TCGA) cohort, and integrated analyses were performed to characterize their mutational landscape, copy number variations, and differential expression profiles. Senescence-related molecular subtypes were established using consensus clustering, followed by gene set variation analysis (GSVA) for pathway enrichment and immune infiltration analyses. A prognostic risk model was subsequently constructed using LASSO-penalized Cox regression, and its predictive performance was systematically evaluated. Candidate key regulators were further prioritized through bioinformatic screening, identifying FOLR1 as a hub gene. The biological function of FOLR1 was validated by qRT–PCR, Western blotting, assessment in clinical specimens, and a subcutaneous xenograft tumor model in mice. **Results:** Cellular senescence-related genes in LUAD exhibited a high frequency of somatic mutations and copy number alterations, accompanied by marked transcriptional dysregulation. Based on the expression profiles of these genes, LUAD patients could be stratified into three distinct molecular subtypes with significantly different clinical outcomes. These subtypes displayed pronounced heterogeneity in pathway enrichment patterns and immune cell infiltration. The subsequently developed prognostic signature demonstrated robust predictive performance in both the training and validation cohorts. Functional assays showed that FOLR1 was significantly downregulated in LUAD tissues and cell lines; FOLR1 knockdown promoted tumor cell proliferation, whereas restoration of its expression or pharmacological intervention markedly suppressed tumor progression. Consistently, *in vivo* xenograft experiments further corroborated the tumor-suppressive role of FOLR1 in lung adenocarcinoma. **Conclusions:** This study systematically delineated the molecular landscape of cellular senescence-related genes in LUAD and elucidated their associations with the tumor immune microenvironment and patient prognosis. Moreover, FOLR1 was identified as a potential tumor suppressor and therapeutic target. These findings provide a theoretical basis for senescence-informed molecular stratification and the development of precision treatment strategies in lung adenocarcinoma.

## Simple Summary

Lung adenocarcinoma is the most common type of lung cancer, but patients often have very different outcomes. This means that better ways are needed to group patients and understand what drives the disease. In this study, we looked at a group of genes linked to a process called cellular senescence, in which damaged cells stop growing but can still affect surrounding tissues. We found that these genes can divide lung adenocarcinoma into different groups with different survival outcomes and tumor features. We also developed a seven-gene score that may help predict prognosis. In addition, we identified FOLR1 as a promising gene of interest, because it was lower in tumor samples and its loss promoted tumor growth in experimental models. These findings may help improve patient classification and guide future studies on lung cancer biology and treatment.

## 1. Introduction

Lung cancer remains one of the most frequently diagnosed malignancies and the leading cause of cancer-related mortality worldwide [1]. Non-small-cell lung cancer (NSCLC) accounts for approximately 85% of all lung cancers, and most patients are diagnosed at an advanced stage, resulting in an unsatisfactory overall 5-year survival rate [2,3]. Lung adenocarcinoma (LUAD) is the most common histological subtype of NSCLC [4]. Recent epidemiological data further underscore the burden of this disease; in the United States, an estimated 229,410 new lung cancer cases and 124,990 deaths are expected in 2026, and adenocarcinoma has become the predominant histological subtype of lung cancer worldwide. Although targeted therapies against epidermal growth factor receptor (EGFR) and anaplastic lymphoma kinase (ALK), as well as immunotherapies such as programmed cell death protein 1 (PD-1)/programmed death-ligand 1 (PD-L1) blockade, have substantially improved outcomes in a subset of patients, marked inter-patient variability in therapeutic response and the high risks of acquired resistance, recurrence, and metastasis remain major clinical challenges [5,6,7]. These observations suggest that conventional histopathological classification or reliance on a limited set of driver alterations is insufficient to capture the biological complexity of LUAD and to meet the demands of precision stratification and individualized treatment [8].

LUAD exhibits profound molecular heterogeneity, which limits the clinical utility of traditional pathological subtyping for guiding diagnosis and therapeutic decision-making [9,10]. Therefore, developing a molecular classification system that better reflects intrinsic tumor biology together with robust, generalizable prognostic models and clinically actionable targets is critical for improving risk stratification and optimizing therapeutic strategies in LUAD [11,12].

Cellular senescence is a stable state of cell-cycle arrest triggered by DNA damage, oxidative stress, or oncogenic signaling [13]. Senescence is accompanied by chromatin remodeling, metabolic reprogramming, altered autophagy, and the acquisition of the senescence-associated secretory phenotype (SASP) [13]. In the context of cancer, senescence exerts dual, context-dependent effects [14]. On the one hand, senescence restricts aberrant proliferation through canonical pathways such as tumor protein p53 (TP53)/cyclin-dependent kinase inhibitor 1A (CDKN1A; p21) and cyclin-dependent kinase inhibitor 2A (CDKN2A; p16^INK4a^)/retinoblastoma 1 (RB1; RB), and is considered a physiological tumor-suppressive response; key oncogenic events can induce senescence in premalignant cells, which may subsequently be eliminated by immune surveillance, thereby limiting tumor progression [15]. On the other hand, the persistent accumulation of senescent cells can promote chronic inflammation and stromal remodeling through SASP, reshaping fibroblast populations, the extracellular matrix (ECM), and immune infiltration, and ultimately facilitating tumor progression and therapeutic resistance [14]. Accordingly, senescence programs may represent a critical nexus linking intrinsic tumor molecular states to heterogeneity within the tumor microenvironment (TME) [15].

In recent years, senescence-associated biomarkers have been explored for prognostic evaluation across multiple cancer types [16]. For example, tumor protein p53 (TP53; p53) immunohistochemical status has been reported to associate with survival outcomes in breast cancer [17]. In addition, basic helix-loop-helix family member e40 (BHLHE40; DEC1) has been implicated in breast cancer progression and is associated with adverse prognosis in clinical datasets [18]. Moreover, the senescence marker tumor necrosis factor receptor superfamily member 10D (TNFRSF10D; DCR2) has been evaluated in breast cancer, including evidence linking DCR2-related alterations (e.g., methylation/expression) with clinicopathological features and outcomes [19,20]. Senescence-associated signatures have also been reported to carry prognostic/predictive relevance in hematologic malignancies (e.g., multiple myeloma) and colorectal cancer [21,22]. In lung cancer, DEC1/BHLHE40-related studies further support links between senescence-associated programs and clinical phenotypes [23]. Nevertheless, systematic senescence gene-based subtyping in LUAD remains limited, with relatively few studies applying consensus clustering based senescence stratification frameworks [24]. In particular, there is still a lack of a coherent body of evidence that can organically connect molecular subtype characteristics, immune microenvironmental states, and predictive models based on key molecules, which to some extent hinders the translation and application of senescence-related research findings in clinical practice [16]. We therefore hypothesized that senescence-related molecular alterations could define biologically and clinically distinct LUAD subgroups, shape pathway activity and tumor microenvironmental states, and be leveraged to construct a robust prognostic framework while uncovering functionally relevant candidate genes.

In this study, we aimed to systematically determine whether senescence-related molecular features could be used to classify LUAD into biologically and prognostically distinct subtypes, to establish a robust prognostic framework, and to identify candidate senescence-associated genes with potential functional and translational relevance [25]. To address this question, we integrated transcriptomic profiles and clinical annotations from The Cancer Genome Atlas (TCGA) and Gene Expression Omnibus (GEO) cohorts to characterize senescence-related molecular alterations in LUAD [26,27]. For transparency, the analytical workflow was separated into a subtype-discovery component based on the batch-corrected combined cohort and a prognostic-modeling workflow consisting of model development in the TCGA-LUAD discovery cohort and external validation in GSE31210 [28,29]. We then performed consensus clustering, pathway analysis, immune infiltration assessment, and prognostic modeling to evaluate the biological and clinical significance of senescence-associated patterns. Finally, key candidate genes identified from the bioinformatic framework were prioritized for experimental validation using cell-based assays, paired clinical specimens, and *in vivo* xenograft models.

## 2. Materials and Methods

### 2.1. Bioinformatic Analyses (The Overall Bioinformatics Workflow Is Summarized in Supplementary Figure S1A)

#### 2.1.1. Data Collection and Preprocessing

All transcriptomic data (RNA sequencing, fragments per kilobase of transcript per million mapped reads FPKM) and corresponding clinical information (including overall survival time, survival status, age, sex, and pathological stage) were downloaded from the lung adenocarcinoma (LUAD) project of The Cancer Genome Atlas (TCGA) and curated/annotated based on the GENCODE reference. Raw expression data were processed in R(4.5.0). Genes with zero expression in >50% of samples were excluded. Ensembl gene identifiers were converted to official gene symbols. The expression matrix was then matched to complete clinical annotations to generate the final TCGA-LUAD cohort for downstream analyses.

The expression matrix and platform annotation of the external validation dataset Gene Expression Omnibus (GEO) accession GSE31210 were retrieved from the Gene Expression Omnibus (GEO) database. Probe identifiers were mapped to gene symbols. Probes annotated to multiple genes were removed; for genes represented by multiple probes, probe intensities were aggregated using the median value. An expression matrix with genes as rows and samples as columns was obtained. The TCGA cohort and the GEO dataset were subsequently merged and batch effects were adjusted using the “sva” package for subsequent analyses. After batch correction, the merged cohort was used for unsupervised senescence-related consensus clustering and DEG-based secondary clustering, whereas the TCGA-LUAD cohort was retained as the discovery cohort for prognostic model development and GSE31210 was reserved for external validation of the final signature.

#### 2.1.2. Differential Expression and Preliminary Prognostic Screening of Senescence-Related Genes

A panel of 33 cellular senescence-related genes was curated from published literature. Differential expression between LUAD tumor tissues and adjacent normal tissues was assessed using the R package “limma”. Genes meeting the criteria of |log2 (fold change)| > 1 and a false discovery rate (FDR) < 0.05 were considered significantly differentially expressed. The expression levels of these differentially expressed senescence-related genes were further evaluated for associations with overall survival using univariable Cox proportional hazards regression, and genes with *p* < 0.05 were retained for model construction.

#### 2.1.3. Construction and Validation of the Prognostic Risk Score Model

Least absolute shrinkage and selection operator-penalized Cox regression (LASSO–Cox) was performed in the TCGA-LUAD discovery cohort to reduce overfitting and identify the most informative prognostic markers using the R package “glmnet”. The optimal penalty parameter (λ) was determined by 10-fold cross-validation. A risk score for each patient was calculated as the weighted sum of gene expression values and corresponding LASSO coefficients using the following formula: Risk score = Σ (expression of gene *i* × coefficient of gene *i*). Patients in the discovery cohort were stratified into high- and low-risk groups according to the median risk score. The locked coefficient formula derived from the discovery cohort was then applied to GSE31210 for external validation, and patients in the validation cohort were dichotomized according to the median risk score within that cohort for survival comparison. Kaplan–Meier survival curves were generated and compared using the log-rank test. Time-dependent receiver operating characteristic (ROC) curve analysis was conducted to calculate the area under the curve (AUC) at 1, 3, and 5 years, thereby evaluating the prognostic performance of the model in both the discovery and validation cohorts.

#### 2.1.4. Somatic Mutation and Copy Number Variation Analyses

Somatic mutation data for LUAD were obtained from TCGA. The R package “maftools” was used to visualize and compare mutation frequency, mutation types, and patterns of mutual exclusivity/co-occurrence in key senescence-related genes between the high- and low-risk groups. Copy number variation (CNV) data from the TCGA-LUAD cohort were analyzed to quantify and display the frequencies of copy number gains and losses affecting key senescence-related genes.

#### 2.1.5. Tumor Immune Microenvironment Analysis

Immune cell infiltration was estimated from gene expression data using the Cell-type Identification By Estimating Relative Subsets Of RNA Transcripts (CIBERSORT) deconvolution algorithm, which quantifies the relative proportions of 22 immune cell subsets in each tumor sample. Differences in immune infiltration abundance between the high- and low-risk groups were subsequently compared.

#### 2.1.6. Functional and Pathway Enrichment Analyses

To explore biological processes associated with the high-risk phenotype, Gene Set Enrichment Analysis (GSEA) was performed on differentially expressed genes between the high- and low-risk groups using the GSEA software (4.4.0). Gene sets were derived from the Molecular Signatures Database (MSigDB), including the Hallmark and Kyoto Encyclopedia of Genes and Genomes (KEGG) collections. Statistical significance was defined as |normalized enrichment score (NES)| > 1, nominal *p* < 0.05, and FDR < 0.25.

### 2.2. Clinical Specimen Collection

Paired lung adenocarcinoma (LUAD) tissues and adjacent non-tumorous lung tissues (*n* = 14 pairs) were collected between June 2025 and December 2025 at Nantong First People’s Hospital, Nantong, China. All specimens were obtained during surgical resection, with an approximate size of 2 × 2 cm for each sample. Prior to RNA extraction, each specimen was divided into two portions for protein extraction and RNA extraction, respectively. This study was approved by the Medical Ethics Committee of Nantong First People’s Hospital (approval No. 2025-KT403), and written informed consent was obtained from all participants.

### 2.3. Cell Lines and Culture Conditions

The normal human bronchial epithelial cell line 16HBE was obtained from the American Type Culture Collection (ATCC, Manassas, VA, USA). Human LUAD cell lines NCI-H1650, NCI-H1975, PC9, HCC827, NCI-H23, and A549 were purchased from the Cell Bank of the Chinese Academy of Sciences (Shanghai, China). 16HBE and A549 cells were maintained in Dulbecco’s modified Eagle’s medium (DMEM, high glucose; Gibco, Thermo Fisher Scientific, Waltham, MA, USA), whereas NCI-H1650, NCI-H1975, PC9, HCC827, and NCI-H23 cells were cultured in Roswell Park Memorial Institute 1640 (RPMI-1640) medium (Gibco, Thermo Fisher Scientific, Waltham, MA, USA). All media were supplemented with 10% fetal bovine serum (FBS, Australian origin; Gibco, Thermo Fisher Scientific, Waltham, MA, USA), 1% penicillin–streptomycin, and 1% L-glutamine (Beyotime Biotechnology, Shanghai, China). Cells were incubated at 37 °C in a humidified atmosphere containing 5% CO_2_.

### 2.4. Lentiviral Vector Construction and Transduction

FOLR1-targeting shRNA lentivirus (sh-FOLR1) and a non-targeting control lentivirus (sh-NC) were designed, constructed, and packaged by GenePharma (Suzhou, China). The supplier used a U6 promoter-driven shRNA lentiviral backbone carrying a puromycin resistance cassette; the lentiviral vectors used in this study did not contain a GFP reporter. The lentiviral titre provided by the manufacturer was >1 × 10^9^ TU/mL.

For transduction, PC9 cells were seeded in 24-well plates at 2 × 10^4^ cells/well and infected with sh-FOLR1 or sh-NC lentivirus at an MOI of 20 in the presence of polybrene (5 μg/mL). At 24 h post-infection, the medium was replaced with fresh complete medium, and stable transductants were selected with puromycin (2 μg/mL) for 1 week. Knockdown efficiency was validated by Western blotting and qRT–PCR.

### 2.5. Western Blot Analysis

Total protein was extracted using radioimmunoprecipitation assay (RIPA) lysis buffer, and protein concentrations were determined using a bicinchoninic acid (BCA) assay. Equal amounts of protein (50 μg per lane) were separated by 10% sodium dodecyl sulfate–polyacrylamide gel electrophoresis (SDS–PAGE) and transferred onto polyvinylidene difluoride (PVDF) membranes. Membranes were blocked with 5% non-fat milk for 2 h at room temperature and then incubated at 4 °C overnight with the following primary antibodies: anti-folate receptor alpha (FOLR1) (rabbit polyclonal, 1:1000; Proteintech, Rosemont, IL, USA; Cat. No. 23355-1-AP), anti-glyceraldehyde-3-phosphate dehydrogenase (GAPDH) (mouse monoclonal, 1:10,000; Proteintech, Rosemont, IL, USA; Cat. No. 60004-1-Ig), and anti-β-actin (ACTB; β-actin) (mouse monoclonal, 1:10,000; Proteintech, Rosemont, IL, USA; Cat. No. 66009-1-Ig). After washing, membranes were incubated with the corresponding secondary antibodies for 2 h at room temperature. Protein bands were visualized using enhanced chemiluminescence (ECL) and quantified by densitometric analysis. FOLR1 protein levels were normalized to GAPDH or β-actin as loading controls.

### 2.6. Quantitative Real-Time PCR

Total RNA was reverse-transcribed into complementary DNA (cDNA) using the PrimeScript RT kit (Takara Bio, Kusatsu, Shiga, Japan). Quantitative real-time polymerase chain reaction (qRT–PCR) was performed using SYBR Green Real-time PCR Master Mix (Takara) according to the manufacturer’s instructions. The thermal cycling program was as follows: initial denaturation at 95 °C for 30 s; 42 cycles of denaturation at 95 °C for 10 s and annealing/extension at 60 °C for 30 s; followed by melt-curve analysis to verify amplification specificity. Relative mRNA expression levels were calculated using the 2^−ΔΔCt^ method, with glyceraldehyde-3-phosphate dehydrogenase (GAPDH) and/or actin beta (ACTB; β-actin) as internal reference genes. Primer sequences for folate receptor alpha (FOLR1), forkhead box M1 (FOXM1), LY6/PLAUR domain containing 3 (LYPD3), polo-like kinase 1 (PLK1), procollagen-lysine,2-oxoglutarate 5-dioxygenase 2 (PLOD2), RAD54-like (RAD54L), STIL centriolar assembly protein (STIL), GAPDH, and ACTB are provided in Supplementary Table S1.

### 2.7. Nude Mouse Xenograft Assay

A subcutaneous xenograft model was established using PC9 cells. The start of the experiment was defined as Day 0. On Day 5, PC9 cells transduced with control shRNA (sh-NC) or folate receptor alpha (FOLR1)-targeting shRNA (sh-FOLR1) were harvested at the logarithmic growth phase, dissociated, and resuspended as single-cell suspensions at 1 × 10^7^ cells/mL. Cells were mixed with Matrigel at the indicated ratio, and 200 μL of the mixture (equivalent to 2 × 10^6^ cells per mouse) was subcutaneously injected into the right axillary region of each nude mouse. After tumor establishment, mice were treated according to group allocation. In the sh-FOLR1 + SG group, sacituzumab govitecan (SG) (10 mg/kg) was administered via tail vein injection (intravenous, i.v.) on Day 12 and Day 19 (two doses in total). During the study period, body weight (and tumor volume, when applicable) was monitored and recorded every 3 days. Mice were euthanized on Day 32; tumors were excised, photographed, and subjected to measurements of tumor volume and weight. Portions of tumor tissues were immediately snap-frozen for subsequent assays, and the remaining tissues were fixed and paraffin-embedded for further analyses.

### 2.8. Statistical Analysis

Statistical analyses and data visualization were performed using GraphPad Prism (version 9.3.1) and R. All *in vitro* experiments were independently repeated at least three times, and data are presented as mean ± standard deviation (SD). For comparisons between two groups, a two-tailed Student’s *t* test was used; if normality or homoscedasticity assumptions were not met, the Wilcoxon rank-sum test was applied. For comparisons among multiple groups, one-way analysis of variance (one-way ANOVA) was performed, followed by post hoc multiple-comparison correction when appropriate. Categorical variables were analyzed using the chi-square (χ^2^) test or Fisher’s exact test, as applicable. Survival analyses were conducted using the Kaplan–Meier method, and differences between groups were assessed by the log-rank test. Correlation analyses were performed using Pearson’s correlation for normally distributed data and Spearman’s correlation for non-normally distributed or ordinal data.

For comparisons of immune cell infiltration across multiple groups (23 cell types), the Kruskal–Wallis test was applied, followed by Benjamini–Hochberg false discovery rate (FDR) correction for multiple comparisons; an adjusted * *p* < 0.05 was considered statistically significant. To assess the biological significance of observed differences, effect sizes (η^2^) were calculated as η^2^ = H/(*n* − 1), where H is the Kruskal–Wallis statistic and *n* is the total number of samples. According to Cohen’s guidelines, η^2^ ≈ 0.01, 0.06, and ≥0.14 were interpreted as small, medium, and large effects. To visualize data overlap across groups, boxplots were overlaid with jittered points; the figure legends specify that boxplots represent the median and interquartile range (IQR), and jittered points indicate individual samples.

All tests were two-sided, and *p* < 0.05 was considered statistically significance. Statistical significance was denoted as * *p* < 0.05, ** *p* < 0.01, *** *p* < 0.001, and **** *p* < 0.0001.

## 3. Results

### 3.1. Genomic Alterations, Copy Number Variation Landscape, and Differential Expression of Senescence-Related Genes in Lung Adenocarcinoma

**Figure 1:**
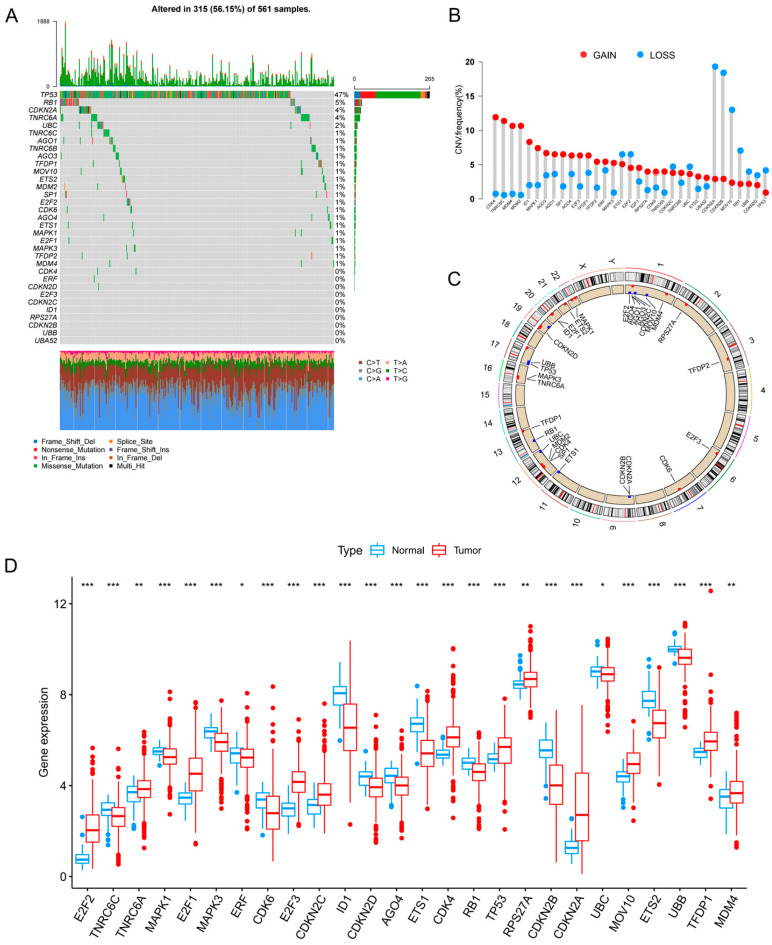
Genomic alterations and expression landscape of senescence-related genes in LUAD. (**A**) Oncoprint of somatic mutations in 33 senescence-related genes in the TCGA-LUAD cohort. The upper bar plot indicates the alteration burden in each sample, and the right panel shows alteration frequencies for each gene. Different colors indicate different mutation types. (**B**) Frequency of copy number gain and loss in the 33 genes. Red indicates gain and blue indicates loss. (**C**) Circos plot showing the chromosomal locations of CNV alterations in the 33 genes. (**D**) Differential expression of senescence-related genes between normal and tumor tissues. Blue indicates normal tissues and red indicates tumor tissues. Statistical significance is indicated as * *p* < 0.05, ** *p* < 0.01, and *** *p* < 0.001.

To systematically characterize the molecular alterations of senescence-related genes in lung adenocarcinoma (LUAD), we performed an integrated genomic and transcriptomic analysis of 33 senescence-associated genes in The Cancer Genome Atlas–lung adenocarcinoma (TCGA-LUAD) cohort. The OncoPrint (2.20.0) summary revealed that 315 of 561 LUAD samples harbored at least one alteration in these genes, corresponding to an overall alteration frequency of 56.15% (Figure 1A), indicating a substantial burden of genomic abnormalities affecting senescence-related genes in LUAD. Copy number variation (CNV) profiling further showed prominent tendencies toward copy number gains (GAIN) or losses (LOSS) across these genes (Figure 1B), and their chromosomal distributions were visualized using a circos plot (Figure 1C). At the transcriptomic level, most senescence-related genes were significantly differentially expressed between tumor and adjacent normal tissues, as illustrated by boxplots depicting distinct expression distributions (Figure 1D). Collectively, these findings demonstrate pervasive genetic alterations and transcriptional dysregulation of senescence-related genes in LUAD, providing a molecular basis for subsequent subtype identification and prognostic model construction.

### 3.2. Consensus Clustering Based on Senescence-Related Genes Identifies Three Molecular Subtypes with Distinct Prognostic Outcomes

**Figure 2:**
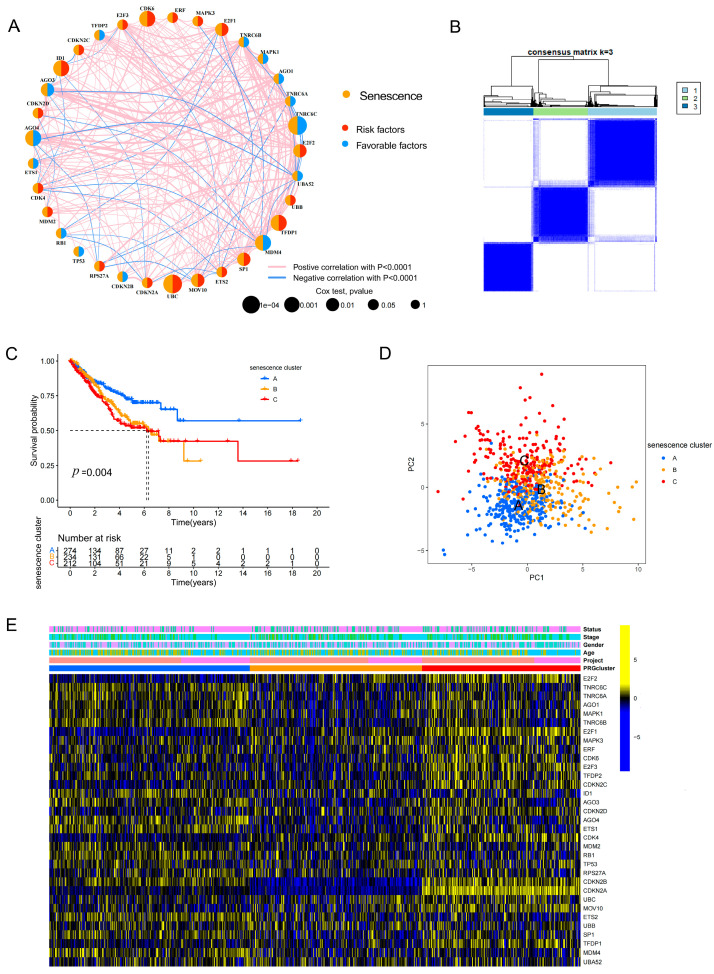
Consensus clustering identifies senescence-related molecular subtypes with distinct prognostic features in LUAD. (**A**) Correlation network of 33 senescence-related genes. Red lines indicate positive correlations and blue lines indicate negative correlations. (**B**) Consensus matrix heatmap showing stable clustering of LUAD samples at *k* = 3. (**C**) Kaplan–Meier overall survival curves of the three senescence clusters. (**D**) Principal component analysis showing the distribution of the three senescence clusters. Partial overlap among clusters is observed, indicating that subtype boundaries are not sharply defined and may reflect a continuum of transcriptomic states. (**E**) Heatmap of the expression patterns of 33 senescence-related genes among the three clusters, with clinical annotations.

Based on the expression profiles of 33 senescence-related genes, we first constructed a gene–gene correlation network (Figure 2A) and subsequently performed consensus clustering to stratify LUAD samples. The consensus matrix indicated that the optimal number of clusters was *k* = 3, enabling robust classification of the merged LUAD cohort (TCGA and GSE31210) into three senescence-associated molecular subtypes (senescence clusters A, B, and C) (Figure 2B). This merged-cohort analysis was used specifically for unsupervised subtype discovery and characterization, thereby distinguishing the clustering step from the subsequent prognostic model training and external validation workflow. Kaplan–Meier survival analysis demonstrated significant differences in overall survival among the three subtypes (*p* = 0.004) (Figure 2C), highlighting the prognostic stratification capacity of this senescence-based classification. Principal component analysis showing the distribution of the three senescence clusters. Although the three clusters show some separation, overlap is observed in the central region, suggesting that subtype boundaries are not entirely discrete and may exhibit continuity. (Figure 2D). A heatmap illustrated systematic differences in senescence-related gene expression patterns across the subtypes, together with clinical annotation tracks (Figure 2E), further supporting the robustness of the classification and its underlying biological heterogeneity.

### 3.3. Distinct Pathway Activities and Immune Microenvironment Landscapes Across Senescence-Associated Subtypes

**Figure 3:**
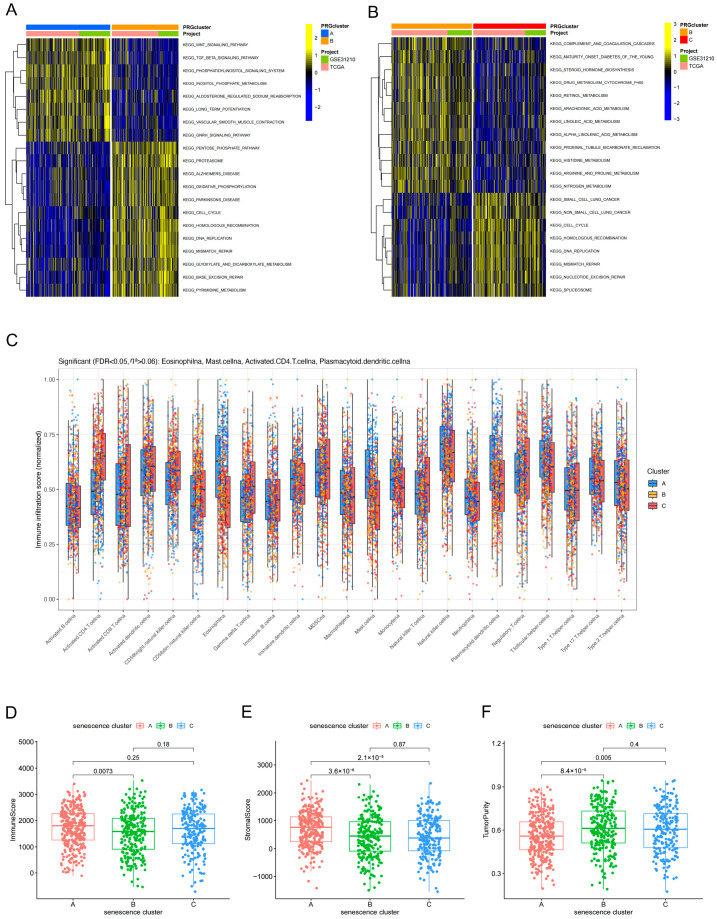
Functional pathway enrichment and immune microenvironment characteristics among senescence subtypes. (**A**,**B**) GSVA heatmaps showing differential KEGG pathway enrichment among the three senescence clusters. (**C**) Comparison of immune cell infiltration among the three clusters based on ssGSEA. The Kruskal–Wallis test followed by Benjamini–Hochberg FDR correction was applied for multiple comparisons (23 cell types). Boxplots display median and interquartile range (IQR); jittered points represent individual samples. (**D**–**F**) Comparison of ImmuneScore (**D**), StromalScore (**E**), and TumorPurity (**F**) among the three senescence clusters. Statistical significance was determined by the Kruskal–Wallis test with FDR correction. *p* values are FDR-adjusted; *p* < 0.05 was considered statistically significant.

To elucidate the potential biological mechanisms underlying the senescence-associated subtypes, we performed gene set variation analysis (GSVA) to quantify subtype-specific pathway activities. The results revealed marked stratification of pathway enrichment patterns across the three subtypes: certain subtypes were preferentially enriched for signaling pathways related to Wnt and transforming growth factor beta (TGF-β), whereas others exhibited higher activity of proliferation-associated programs, including cell cycle regulation and DNA replication/repair (Figure 3A,B). With respect to immune infiltration, single-sample gene set enrichment analysis (ssGSEA) indicated significant differences in the infiltration levels of multiple immune cell populations among the three subtypes (Figure 3C). Further quantitative assessment of the tumor microenvironment showed that ImmuneScore, StromalScore, and tumor purity also differed significantly across subtypes (Figure 3D–F). Collectively, these findings suggest that the senescence-based classification not only captures intrinsic biological programs within tumors but is also closely associated with the immune microenvironmental state.

### 3.4. Secondary Clustering Based on Subtype-Associated Differentially Expressed Genes Further Refines Prognostic Stratification

**Figure 4:**
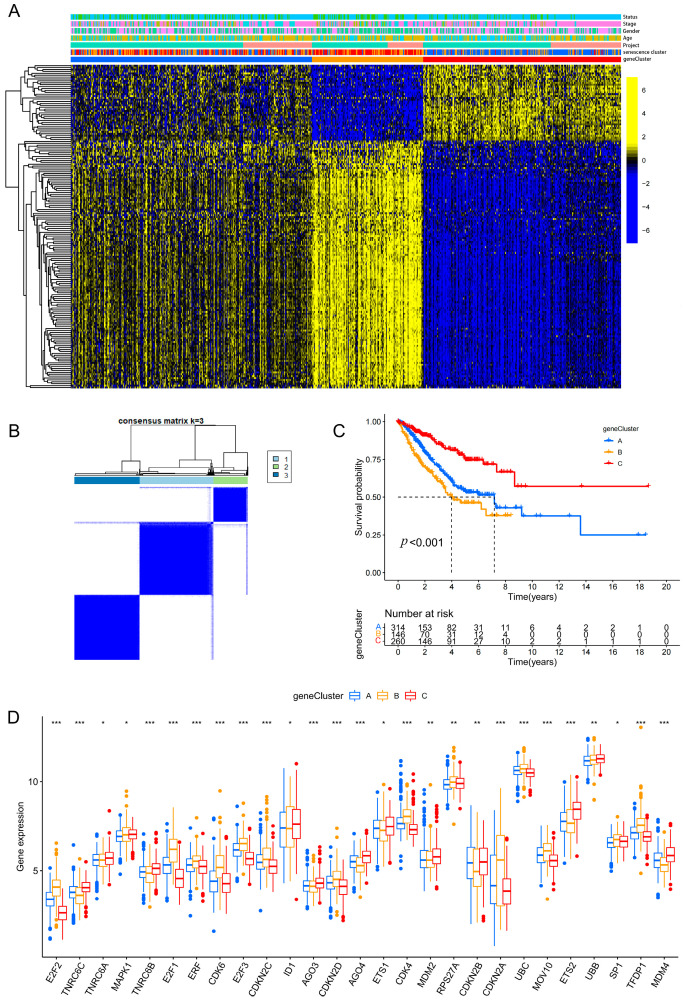
Secondary clustering based on DEGs further refines prognostic stratification in LUAD. (**A**) Heatmap of unsupervised clustering based on subtype-associated DEGs, with annotations of clinical features, senescence clusters, and geneClusters. (**B**) Consensus matrix showing stable clustering of geneCluster A, B, and C at k = 3. (**C**) Kaplan–Meier overall survival curves of the three geneClusters. (The number of patients at risk is shown below the plot; survival estimates beyond 60 months should be interpreted with caution due to reduced sample size.) (**D**) Differential expression of senescence-related genes among geneClusters. Statistical significance is indicated as * *p* < 0.05, ** *p* < 0.01, *** *p* < 0.001.

Building on the senescence-based subtyping framework, we performed a secondary consensus clustering analysis using the differentially expressed genes (DEGs) associated with the senescence subtypes, yielding three DEG-driven clusters (geneClusters A, B, and C) (Figure 4A,B). Kaplan–Meier analysis demonstrated a highly significant difference in overall survival among the geneClusters (*p* < 0.001) (Figure 4C), indicating that this DEG-based secondary classification further refines outcome stratification beyond the initial senescence clusters. In parallel, the expression levels of senescence-related genes differed markedly across the geneClusters (Figure 4D), suggesting that the geneClusters preserve senescence-associated biological features while enhancing discrimination of clinical prognosis.

### 3.5. Development and Validation of a Seven-Gene Prognostic Risk Signature with Robust Survival Prediction and Immune-Associated Differences

**Figure 5:**
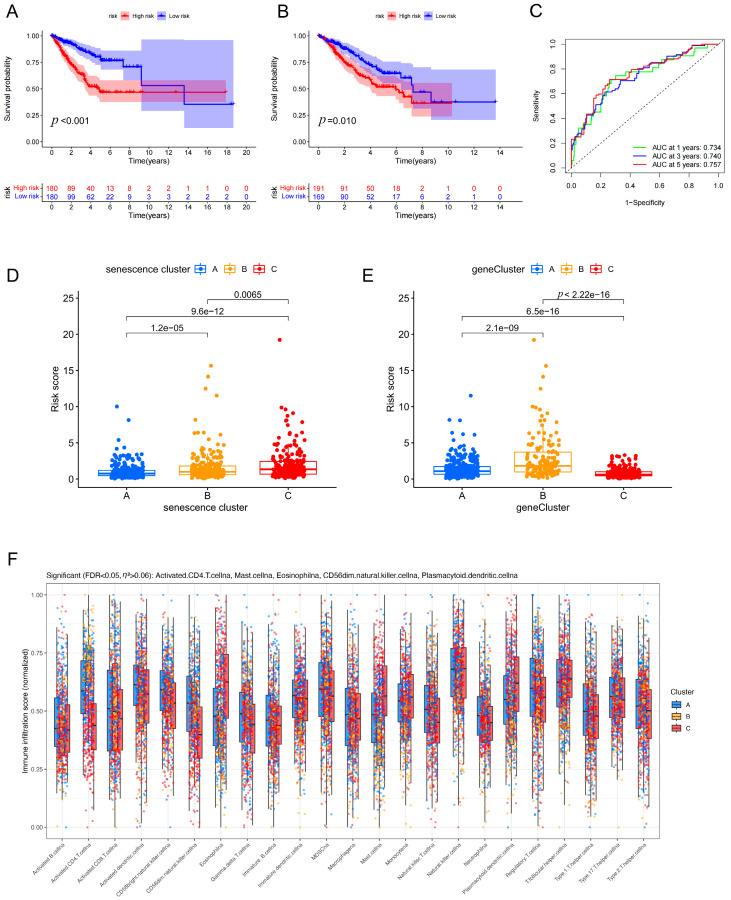
Construction and validation of a 7-gene prognostic model in LUAD. (**A**) Kaplan–Meier overall survival curves of high- and low-risk groups in the TCGA-LUAD discovery cohort. (**B**) Kaplan–Meier overall survival curves of high- and low-risk groups in the independent GSE31210 validation cohort. (**C**) Time-dependent ROC curves showing the predictive performance of the 7-gene risk model at 1, 3, and 5 years. (**D**) Comparison of risk scores among the three senescence clusters. (**E**) Comparison of risk scores among the three geneClusters. (**F**) Comparison of immune cell infiltration among different geneClusters. Bootstrap internal validation metrics are summarized in Supplementary Table S3.

To clarify the prognostic workflow, we used the TCGA-LUAD cohort as the discovery cohort for model construction and the independent GSE31210 dataset as the external validation cohort. The gene selection process is summarized in Supplementary Figure S1B. Briefly, from 33 senescence-related genes, we sequentially applied differential expression analysis (|log_2_FC| > 1, FDR < 0.05), univariate Cox regression (*p* < 0.05), and LASSO-Cox regression with 10-fold cross-validation. A total of 15 prognosis-related genes entered the LASSO model, and seven genes with non-zero coefficients were finally selected. Based on the key subtype-associated differential features, we constructed a prognostic risk score model comprising seven genes: folate receptor alpha (FOLR1), forkhead box M1 (FOXM1), LY6/PLAUR domain containing 3 (LYPD3), polo-like kinase 1 (PLK1), procollagen-lysine,2-oxoglutarate 5-dioxygenase 2 (PLOD2), RAD54-like (RAD54L), and STIL centriolar assembly protein (STIL). The coefficient formula established in the discovery cohort was subsequently applied un-changed to the validation cohort. In the discovery cohort, Kaplan–Meier analysis showed that patients in the high-risk group had significantly worse overall survival than those in the low-risk group (*p* < 0.001) (Figure 5A). Consistently, in the external validation cohort, high-risk patients also exhibited inferior survival outcomes (*p* = 0.010) (Figure 5B), supporting the robustness and generalizability of the signature. Bootstrap internal validation with 100 resamples yielded a mean C-index of 0.674 (95% CI: 0.626–0.714), which was comparable to the original training C-index of 0.668, further confirming the stability of the prognostic signature and arguing against severe overfitting. Time-dependent receiver operating characteristic (ROC) analysis demonstrated favorable predictive performance, with areas under the curve (AUCs) of 0.734, 0.740, and 0.757 at 1, 3, and 5 years, respectively (Figure 5C). In addition, risk scores differed significantly across senescence clusters and geneClusters (Figure 5D,E). The robustness of the risk score differences was confirmed by outlier sensitivity analysis and effect size calculations. Briefly, excluding 16 extreme values (2.2% of samples) did not materially alter the significance or effect size, and multiple effect size measures (η^2^ = 0.1685, Cohen’s d = 0.608–0.942) indicated moderate-to-large effects (detailed in Supplementary Table S2) Moreover, immune infiltration landscapes varied among geneClusters (Figure 5F), suggesting that risk stratification is, at least in part, associated with distinct tumor immune microenvironmental features.

### 3.6. Single-Gene Prognostic Analyses Identify FOLR1 as a Protective Factor, with In Vitro Validation of Its Expression Pattern and Establishment of an Intervention Model

**Figure 6:**
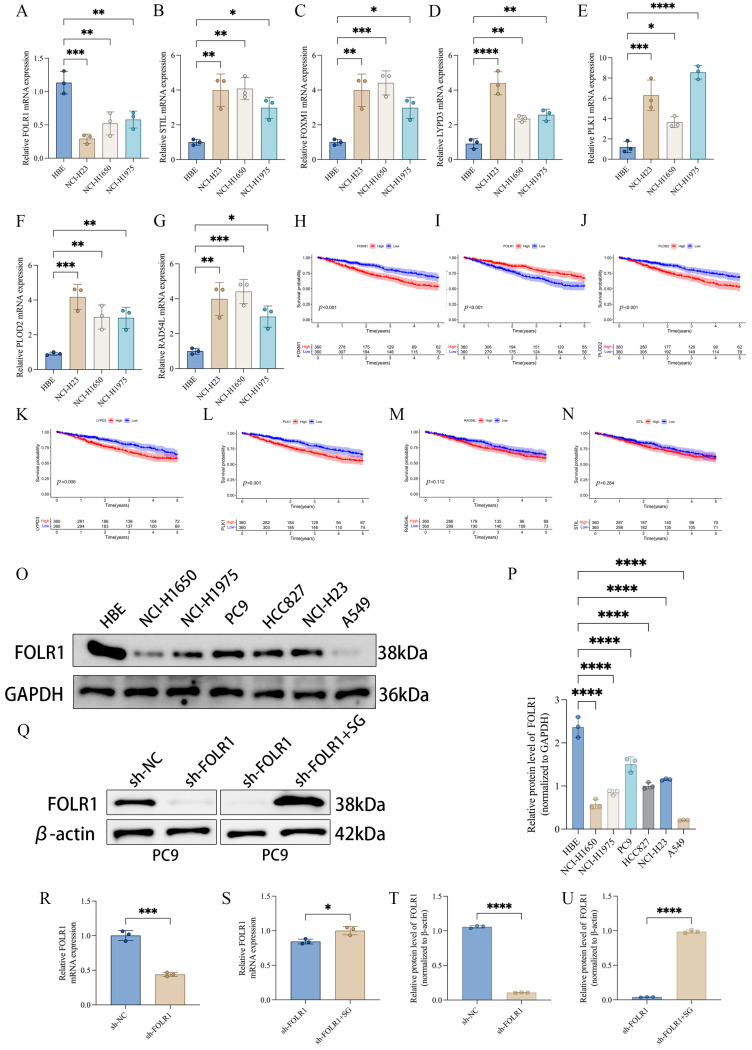
FOLR1 acts as a potential protective factor in LUAD. (**A**–**G**) qRT-PCR analysis of the expression of seven key genes in HBE cells and multiple lung cancer cell lines. (**H**–**N**) Kaplan–Meier survival analyses showing the associations between the seven key genes and overall survival in LUAD. (**O**,**P**) Western blot and densitometric analysis of FOLR1 protein expression in HBE cells and lung cancer cell lines. (**Q**) Establishment of the FOLR1 intervention model in PC9 cells (sh-NC, sh-FOLR1, and sh-FOLR1 + SG), with Western blot validation. (**R**–**U**) qRT-PCR and protein quantification confirming the changes in FOLR1 expression after intervention. SG, Sacituzumab govitecan. Statistical significance is indicated as * *p* < 0.05, ** *p* < 0.01, *** *p* < 0.001, **** *p* < 0.0001.

Among the seven genes included in the prognostic signature, we performed single-gene survival analyses to prioritize candidates with independent prognostic relevance. The results showed that high expression of forkhead box M1 (FOXM1) and procollagen-lysine,2-oxoglutarate 5-dioxygenase 2 (PLOD2) was associated with poorer survival, whereas high expression of folate receptor alpha (FOLR1) predicted favorable clinical outcomes (Figure 6H–N), suggesting that FOLR1 may act as a protective factor in LUAD. In vitro, quantitative real-time polymerase chain reaction (qRT–PCR) revealed that FOLR1 expression was generally reduced across multiple lung cancer cell lines compared with the normal human bronchial epithelial cell line 16HBE. In contrast, the remaining signature genes—FOXM1, LY6/PLAUR domain containing 3 (LYPD3), polo-like kinase 1 (PLK1), PLOD2, RAD54-like (RAD54L), and STIL centriolar assembly protein (STIL)—exhibited an overall upregulated trend in lung cancer cell lines (Figure 6A–G). Consistently, analysis of the TCGA-LUAD cohort showed that FOLR1 expression was significantly lower in tumor tissues than in adjacent normal tissues (Supplementary Figure S1C). Western blotting further confirmed decreased FOLR1 protein expression in several lung cancer cell lines relative to 16HBE (Figure 6O,P). A stable FOLR1-knockdown model was subsequently established in PC9 cells, in which FOLR1 protein levels were markedly reduced in the sh-FOLR1 group. Notably, upon treatment with sacituzumab govitecan (SG) in the knockdown background (sh-FOLR1 + SG), FOLR1 protein levels showed a partial restoration/rebound trend (Figure 6Q). Consistently, qRT–PCR measurements at the mRNA level and quantitative protein densitometry analyses corroborated these findings (Figure 6R–U). Collectively, these results support the protective association of FOLR1 from both clinical prognostic and experimental expression/intervention perspectives, providing a rationale for subsequent *in vivo* validation.

### 3.7. Validation in Clinical Specimens and Xenograft Models: Low FOLR1 Expression Is Associated with a Pro-Tumorigenic Phenotype and Is Partially Reversed by SG Treatment

**Figure 7:**
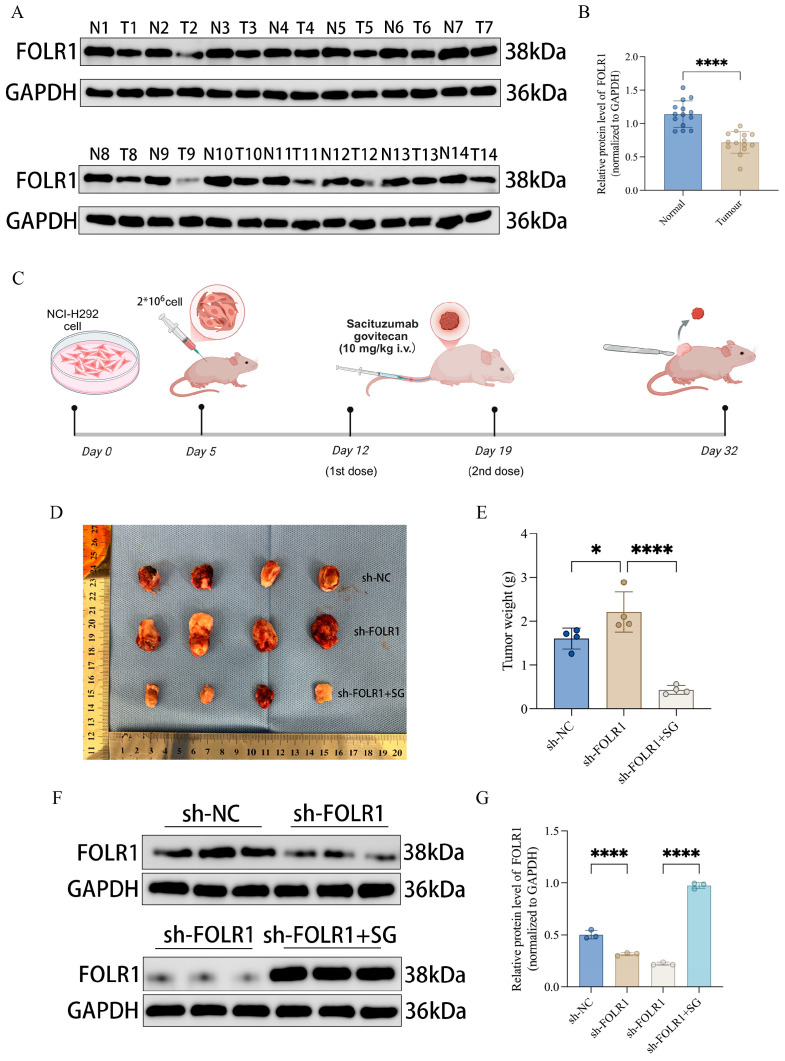
Clinical and *in vivo* validation of FOLR1 in LUAD. (**A**) Western blot analysis of FOLR1 in paired adjacent normal and tumor tissues. (**B**) Densitometric quantification of FOLR1 protein levels in paired clinical samples. (**C**) Schematic diagram of the PC9 xenograft experiment. (**D**) Representative images of xenograft tumors from the sh-NC, sh-FOLR1, and sh-FOLR1 + SG groups. (**E**) Tumor weight comparison among groups. (**F**) Western blot analysis of FOLR1 protein expression in xenograft tissues. (**G**) Densitometric quantification of FOLR1 protein expression in xenograft tissues. Statistical significance is indicated as * *p* < 0.05, **** *p* < 0.0001.

To validate the expression pattern of folate receptor alpha (FOLR1) in clinical tissues, we performed Western blot analysis on paired specimens. FOLR1 protein levels were lower in tumor tissues (T) than in the matched adjacent non-tumorous tissues (N), and quantitative densitometry confirmed a statistically significant reduction in tumors (Figure 7A,B). We next evaluated the functional relevance of FOLR1 *in vivo* using a subcutaneous xenograft model established with PC9 cells, with interventions implemented according to the experimental timeline (Figure 7C). Briefly, on Day 5, mice were subcutaneously inoculated with PC9 cells; in the sh-FOLR1 + SG group, sacituzumab govitecan (SG) (10 mg/kg) was administered via tail vein injection (intravenous, i.v.) on Day 12 and Day 19, and tumors were harvested on Day 32. Representative images of excised tumors showed increased tumor burden in the sh-FOLR1 group, whereas SG treatment reduced tumor size in the knockdown background (Figure 7D). Consistently, tumor weight measurements indicated that sh-FOLR1 tumors were heavier than those in the control group, while tumors in the sh-FOLR1 + SG group were significantly lighter (Figure 7E). These data suggest that SG treatment can partially reverse the enhanced tumor growth phenotype induced by FOLR1 depletion. Western blot analyses of xenograft tissues and corresponding quantification further demonstrated that changes in FOLR1 protein levels across treatment groups were concordant with the observed phenotypic differences (Figure 7F,G). Taken together, FOLR1 is downregulated in LUAD, and its reduction is associated with augmented tumor growth. Importantly, SG treatment markedly suppressed tumor growth *in vivo* and was accompanied by a trend toward restoration of FOLR1 protein expression.

## 4. Discussion

In this study, we established an integrated workflow encompassing senescence-associated molecular characterization, molecular subtyping, prognostic modeling, key gene validation, and *in vivo* pharmacological intervention to systematically delineate the multidimensional alterations of senescence-related genes in lung adenocarcinoma (LUAD) and their clinical relevance [30]. At both the genomic and transcriptomic levels, senescence-related genes exhibited pervasive somatic alterations, copy number variation (CNV) abnormalities, and transcriptional dysregulation (Figure 1), suggesting that senescence programs are not merely confined to isolated pathway perturbations but may represent broadly remodeled biological events during LUAD initiation and progression. Based on these observations, consensus clustering robustly classified patients into three senescence-associated molecular subtypes with significantly different survival outcomes (Figure 2), indicating that senescence-related expression patterns capture clinically meaningful heterogeneity and provide an interpretable molecular framework for risk stratification in LUAD.

Notably, senescence-based subtyping was not only prognostically informative but also accompanied by distinct pathway activities and tumor microenvironmental features (Figure 3). Rather than reflecting only generalized senescence-related concepts, the subtype differences observed in our dataset point to discrete biological states. Specifically, the GSVA results showed that one subtype was relatively enriched for proliferation/replicative-stress programs, including cell cycle and DNA replication/repair [30,31], whereas another subtype was more closely associated with Wnt/TGF-β signaling and extracellular matrix remodeling, supporting a stromal-remodeling phenotype (Figure 3A,B). In parallel, the immune-related findings in this study were directly based on the observed differences in immune cell infiltration, ImmuneScore, StromalScore, and tumor purity across senescence clusters (Figure 3C–F), rather than on general assumptions about SASP biology alone. In this context, SASP-, stromal-, and immune-related processes are most relevant as interpretive frameworks for the subtype characterized by Wnt/TGF-β signaling, extracellular matrix remodeling, and an immune-/stroma-enriched microenvironment, whereas the more proliferation-dominant subtype may represent a distinct replicative-stress state with different clinical behavior [32,33]. Thus, our data support the view that senescence-associated subtype heterogeneity in LUAD is linked to concrete differences in pathway activity and tumor microenvironmental composition, which may contribute to variation in prognosis and treatment response. These subtype-specific observations provide a more direct basis for discussing SASP, stromal remodeling, and immune-related alterations in the context of the present study [34].

Building on the primary senescence subtypes, we performed secondary clustering based on subtype-associated differentially expressed genes (DEGs) to derive geneClusters, which further strengthened prognostic discrimination (Figure 4). This observation suggests that the senescence-based classifier may capture upstream core programs, whereas DEG-driven geneClusters could more closely represent downstream effector networks and integrated tumor phenotypes, thereby offering greater sensitivity for outcome prediction [35]. From a translational perspective, a hierarchical stratification strategy combining “primary senescence subtypes secondary DEG-based subtypes” may improve patient stratification granularity and provide a refined basis for risk-adapted surveillance, individualized treatment, and clinical risk management.

Furthermore, the seven-gene prognostic signature developed in this study demonstrated stable predictive performance in both the overall cohort and the validation cohort (Figure 5A–C), and the risk score distribution was concordantly associated with both senescence clusters and geneClusters (Figure 5D,E). The clarified discovery-to-validation workflow, with model development in TCGA-LUAD and external assessment in GSE31210, improves the transparency and interpretability of the prognostic analysis. Although the prognostic significance was attenuated in the validation cohort (*p* = 0.01) compared to the training cohort (*p* < 0.001), bootstrap internal validation excluded severe overfitting as the primary cause (bootstrap C-index: 0.674 vs. original: 0.668). The reduced effect size in the validation set is more likely attributable to cohort heterogeneity, including differences in platform (RNA-seq vs. microarray), sample size, and clinical characteristics. Importantly, these results argue against a purely “black-box” statistical fit; instead, the risk model is biologically anchored to upstream senescence-associated subtypes and their molecular differences, thereby enhancing interpretability and potential portability across cohorts [30]. In addition, differences in immune infiltration patterns among gene Clusters (Figure 5F) suggest that the risk score may partially reflect the immune microenvironmental state. Future work integrating the risk signature with immunological features such as immune scores, T-cell inflamed signatures, or tumor mutational burden may further improve prediction of immunotherapy response or recurrence risk [36].

At the individual-gene level, single-gene survival analyses identified folate receptor alpha (FOLR1), forkhead box M1 (FOXM1), and procollagen-lysine,2-oxoglutarate 5-dioxygenase 2 (PLOD2) as significantly associated with prognosis. High FOXM1 and PLOD2 expression predicted unfavorable outcomes, whereas high FOLR1 expression was associated with improved survival (Figure 6H–N). FOXM1 is a canonical driver of proliferation and cell-cycle progression, and its upregulation is consistent with malignant progression and enhanced replicative stress. PLOD2 is tightly linked to collagen crosslinking and stromal remodeling, and elevated PLOD2 expression is frequently associated with increased invasiveness and poor prognosis. In contrast, FOLR1 was downregulated in lung cancer cell lines and clinical tumor specimens (Figure 6A–P; Figure 7A,B), yet higher FOLR1 expression correlated with better survival, suggesting that FOLR1 may represent a candidate tumor-suppressive-associated molecular feature in LUAD rather than a confirmed senescence regulator. This finding offers a complementary perspective to the prevailing view of FOLR1 as a tumor-associated target and underscores that the biological significance of FOLR1 may be context dependent, varying across tumor types, molecular backgrounds, or microenvironmental states. Given that FOLR1 was identified within a senescence-related analytical framework, these observations raise the possibility that FOLR1 may be linked to senescence-associated tumor biology. However, the current evidence does not establish a direct mechanistic role for FOLR1 in canonical senescence pathways. At present, our inference is primarily supported by expression–outcome associations and phenotypic consequences of functional perturbation, whereas the underlying molecular mechanisms remain to be clarified. Further studies examining senescence-associated cell-cycle arrest, canonical senescence markers, SASP-related alterations, and DNA damage response signaling will be necessary to determine whether FOLR1 directly contributes to senescence regulation in LUAD [37].

A key strength of this study is the combined *in vitro*/*in vivo* validation of FOLR1 and the incorporation of pharmacological intervention [38]. Using a stable shRNA-mediated knockdown model in PC9 cells, we observed enhanced tumor growth upon FOLR1 depletion, whereas treatment with sacituzumab govitecan (SG) significantly reduced tumor weight and was accompanied by a trend toward restoration of FOLR1 protein levels (Figure 7D–G). These results functionally support an association between reduced FOLR1 and a pro-tumorigenic phenotype, while also indicating an anti-tumor effect of SG in this model. However, these functional findings primarily support a tumor-suppressive–associated role of FOLR1 and do not, by themselves, demonstrate that FOLR1 directly regulates senescence pathways [39]. Moreover, the clearly defined *in vivo* timeline and dosing regimen (SG, 10 mg/kg, intravenous administration on Day 12 and Day 19) (Figure 7C) enhances reproducibility and provides a foundation for future studies evaluating dose response relationships, regimen optimization, and combination strategies. Nevertheless, the observation that “SG treatment coincides with increased FOLR1 protein levels” should be interpreted cautiously. Whether this represents direct regulation of FOLR1 or a secondary consequence of reduced tumor burden cannot be concluded from the current data. Future experiments assessing the direct effects of SG on FOLR1 transcription/translation *in vitro*, and validating consistency across additional cell lines with distinct baseline FOLR1 expression, will be important to establish causality and define the applicable context.

**Figure 8:**
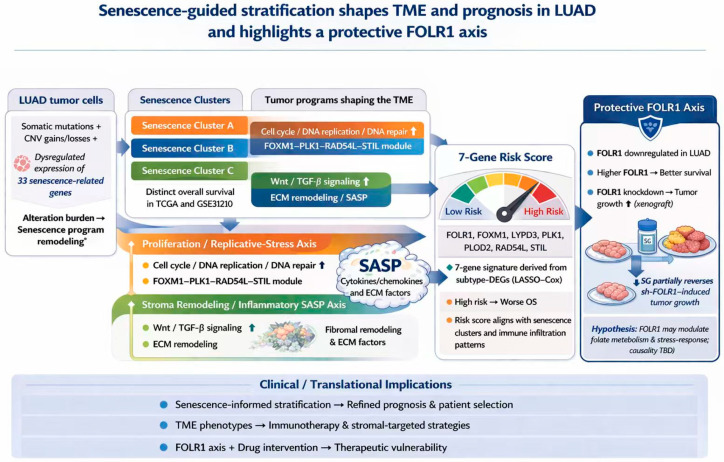
Schematic summary of senescence-guided molecular stratification and the candidate protective role of FOLR1 in LUAD. Multi-omic dysregulation of 33 senescence-related genes defines three senescence-associated clusters in LUAD with distinct survival outcomes. These clusters are linked to proliferation/replicative-stress and stroma remodeling/inflammatory SASP programs. A subtype-DEG-derived 7-gene LASSO–Cox signature yields a risk score associated with immune infiltration and poor prognosis. FOLR1 is highlighted as a candidate protective axis, showing reduced expression in LUAD, favorable prognostic association at higher expression levels, and increased xenograft growth after depletion, partially reversed by SG. This schematic summarizes the principal findings and their potential biological and translational relevance in LUAD.

Despite the relatively comprehensive evidence chain, several limitations should be acknowledged. First, the subtyping framework and risk model were primarily derived from public cohorts; although validated, larger multicenter clinical cohorts are still required to confirm stability and generalizability across populations, sequencing platforms, and clinical settings [40]. In particular, validation in larger and more diverse cohorts would further strengthen the robustness and generalizability of the present findings. Second, the association between senescence subtypes and immune infiltration currently relies largely on computational inference (e.g., ssGSEA and ESTIMATE). Experimental validation using multiplex immunohistochemistry, flow cytometry, or spatial transcriptomics would strengthen the mechanistic credibility by confirming key immune subsets and their spatial distributions. Therefore, the current immune-related observations should be interpreted with caution, as direct experimental validation of the immunological analyses is still lacking. Third, the molecular mechanisms underlying the protective role of FOLR1 remain unresolved, particularly its relationships with senescence programs, SASP composition, metabolic reprogramming, and DNA damage responses. More rigorous functional studies, including rescue experiments and assays of senescence markers, autophagy, reactive oxygen species, and DNA damage response axes, are required to establish mechanistic links [41]. In addition, clinicopathological annotations were not uniformly complete across cohorts, which limited comprehensive multivariable evaluation of the risk score together with conventional clinical variables; this issue should be addressed in future validation studies with harmonized clinical data. To facilitate an integrated interpretation of the present findings, a final schematic model is provided in Figure 8, summarizing senescence-associated molecular alterations, subtype heterogeneity, prognostic stratification, and the candidate protective role of FOLR1 within a senescence-related framework.

In summary, our study demonstrates that senescence-related gene signatures effectively capture LUAD molecular heterogeneity and enable robust prognostic stratification through both subtype classification and a seven-gene risk model. Moreover, we identified FOLR1 as a prognostically favorable candidate gene that is downregulated in LUAD and functionally associated with tumor growth suppression in experimental models. Although these findings suggest that FOLR1 may represent a candidate tumor-suppressive factor identified within a senescence-related framework, its direct mechanistic involvement in senescence pathways remains to be established. Together, these findings provide a basis for further investigation into senescence-associated molecular stratification and candidate therapeutic targets in LUAD.

## 5. Conclusions

Senescence-related molecular features can stratify LUAD into biologically and prognostically distinct subtypes and support the development of a robust seven-gene prognostic model. FOLR1 emerged as a prognostically favorable candidate gene that is downregulated in LUAD and functionally associated with suppression of tumor growth in experimental models. However, its direct mechanistic link to canonical senescence pathways remains unresolved. Overall, these findings support the value of senescence-informed molecular stratification in LUAD and provide a rationale for future mechanistic studies of FOLR1 and other candidate genes.

## Data Availability

Publicly available datasets analyzed in this study can be found in TCGA and GEO. The experimental and clinical data generated during the current study are available from the corresponding author upon reasonable request.

## Supplementary Materials

The following supporting information can be downloaded at: https://www.mdpi.com/article/10.3390/cancers18091330/s1, Figure S1: Workflow of senescence-related molecular subtyping, prognostic modeling, and downstream association analyses in LUAD; Table S1: qPCR Primer Information Sheet; Table S2: Robustness analysis of risk score differences across senescence clusters; Table S3: Internal Validation Metrics of the Prognostic Model.
