# Supplementary material for: Integrative Analysis of Cellular Senescence-Related Genes Identifies FOLR1 as a Novel Tumor Suppressor and a Potential Therapeutic Target in Lung Adenocarcinoma
Source: Cancers (Basel). 2026 Apr 22;18(9):1330. doi: 10.3390/cancers18091330 (PMC13162693; doi:10.3390/cancers18091330)
Supplement: Supplementary file 1 [file cancers-18-01330-s001.zip › Supplementary Table S1.pdf]

Supplementary Table S1. qPCR Primer Information Sheet

Primer sequences are shown in the 5′→3′ direction

Gene	Forward primer （5′→3′）	Reverse primer （5′→3′）
**FOLR1**	CTTGCTGGTGTTGGTAGAACAG	CAGGTTCTGGGCTGTGATGT
**FOXM1**	GCGACAGGTTAAGGTTGAG	GAGGTTGTGGCGGATGG
**LYPD3**	ACCAATGTGTGTGCTTCTGG	GGACTGCTGAGGACTTCTGC
**PLK1**	TCAAGTCCACCCAGAGTGTAA	GTTGCAGTCCAAAGAGGCA
**PLOD2**	TTGGTCCAGTACAACTGCTACA	TGTGTACGGAAGGCTGGTTG
**RAD54L**	GTTGGAAATGGATGTTGTTGC	GAGTCGTTTGCTATTTCCACC
**STIL**	AGCCTGTTTCTGCTGCTGAT	TCCAGGTTGATGTTGGTGGT
**GAPDH**	CCTGGATACCGCAGCTAGGA	GCGGCGCATACGAATGCCCC
**β-actin**	TTGCCGACAGGATGCAGAAGGA	AGGTGGACAGCGAGGCCAGGAT
