# Supplementary material for: Integrative Analysis of Cellular Senescence-Related Genes Identifies FOLR1 as a Novel Tumor Suppressor and a Potential Therapeutic Target in Lung Adenocarcinoma
Source: Cancers (Basel). 2026 Apr 22;18(9):1330. doi: 10.3390/cancers18091330 (PMC13162693; doi:10.3390/cancers18091330)
Supplement: Supplementary file 1 [file cancers-18-01330-s001.zip › Supplementary Table S2.pdf]

Supplementary Table S2. Robustness analysis of risk score differences across senescence clusters

Summary metrics from the primary, sensitivity, and effect-size analyses.

Analysis	Statistic	Value
**Main analysis**	Kruskal–Wallis p-value	**8.32×10^{-31}**
	Eta-squared (η^2)	**0.1685**
**Outlier sensitivity analysis**	Kruskal–Wallis p-value (after removing 16 extreme values)	**1.10×10^{-26}**
	Eta-squared (after removal)	**0.1649**
**Pairwise Cohen's d**	Cluster A vs. B	**0.608**
	Cluster A vs. C	**0.942**
	Cluster B vs. C	**0.712**
**Cohen's f**	Cohen's f	**0.45**
**Rank-biserial correlation (r)**	Range	**0.52–0.68**

Abbreviations: η^2, eta-squared; CI, confidence interval.
