# Supplementary material for: Integrative Analysis of Cellular Senescence-Related Genes Identifies FOLR1 as a Novel Tumor Suppressor and a Potential Therapeutic Target in Lung Adenocarcinoma
Source: Cancers (Basel). 2026 Apr 22;18(9):1330. doi: 10.3390/cancers18091330 (PMC13162693; doi:10.3390/cancers18091330)
Supplement: Supplementary file 1 [file cancers-18-01330-s001.zip › Supplementary Table S3.pdf]

Supplementary Table S3. Internal Validation Metrics of the Prognostic Model

Metric	Value
Original C-index (training set)	0.668
Bootstrap mean C-index (100 resamples)	0.674
Bootstrap 95% confidence interval	0.626–0.714
