# Supplementary figures and images for: Integrative Analysis of Cellular Senescence-Related Genes Identifies FOLR1 as a Novel Tumor Suppressor and a Potential Therapeutic Target in Lung Adenocarcinoma
Source: Cancers (Basel). 2026 Apr 22;18(9):1330. doi: 10.3390/cancers18091330 (PMC13162693; doi:10.3390/cancers18091330)

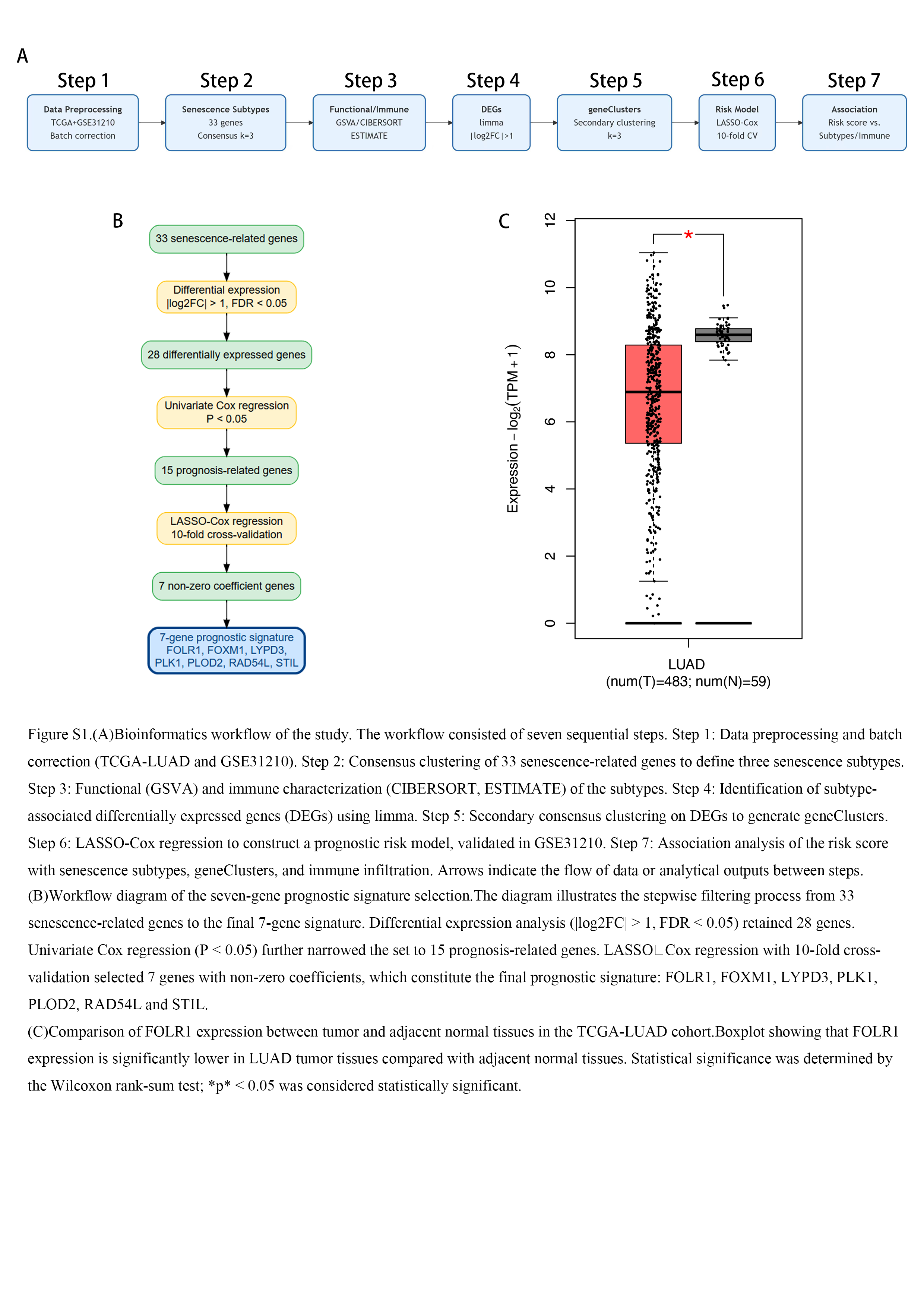

Supplement: Supplementary file 1 [file cancers-18-01330-s001.zip › Supplementary Figure S1.tif]
